# Triple reversal phenomenon in EGFR-mutant lung adenocarcinoma with prostate metastasis following hepatocellular carcinoma: a rare Case Report with diagnostic and therapeutic implications

**DOI:** 10.3389/fmed.2025.1619466

**Published:** 2025-07-16

**Authors:** Jieyan Luo, Jie Zhou, Li Liu

**Affiliations:** Cancer Center, Ziyang Central Hospital, Ziyang, Sichuan, China

**Keywords:** case report, EGFR mutation, lung adenocarcinoma, multidisciplinary approach, prostate metastasis, targeted therapy, triple reversal phenomenon

## Abstract

**Background:**

Non-small cell lung cancer harboring EGFR mutations is responsive to targeted therapies such as Osimertinib. Although metastasis from lung cancer to the prostate is exceedingly rare, we present a rare case of prostatic metastasis from lung adenocarcinoma in a patient with a history of hepatocellular carcinoma (HCC) and no evidence of a primary lung lesion.

**Case Presentation:**

A 64-years-old male with chronic hepatitis B and a history of hepatocellular carcinoma (HCC) diagnosed in 2014 presented in 2023 with elevated carcinoembryonic antigen (CEA) levels. Initial imaging revealed isolated bone metastasis, initially presumed to be recurrent HCC. Given the long interval since diagnosis, a bone biopsy was performed, unexpectedly showing adenocarcinoma. Subsequent PET-CT identified a prostatic lesion without pulmonary abnormalities, leading to an initial diagnosis of metastatic prostate cancer. Prostate biopsy, however, revealed features consistent with lung adenocarcinoma. Molecular testing detected an EGFR exon 21 L858R mutation, confirming metastatic lung adenocarcinoma. The patient responded favorably to osimertinib therapy.

**Conclusion:**

This case illustrates a rare instance of prostatic metastasis from EGFR-mutant lung adenocarcinoma and emphasizes the critical role of repeat biopsy, molecular profiling, and multidisciplinary evaluation in atypical metastatic presentations. The diagnostic process involved a “triple reversal” phenomenon, revising initial misdiagnoses of recurrent HCC and primary prostate cancer to metastatic NSCLC. Targeted therapy with osimertinib was effective, underscoring the importance of precision oncology in managing complex metastatic disease.

## Highlights

- Prostatic metastasis from lung adenocarcinoma is a rare and diagnostically challenging entity.
- The patient underwent three diagnostic revisions: presumed HCC recurrence → metastatic prostate cancer → EGFR-mutant lung adenocarcinoma.
- Repeat biopsy of the metastatic site was instrumental in clarifying the diagnosis.
- EGFR exon 21 L858R mutation confirmed the pulmonary origin of the adenocarcinoma.
- Targeted therapy with Osimertinib yielded favorable therapeutic outcomes.
- Comprehensive multidisciplinary evaluation is essential in cases with atypical metastatic patterns and prior cancer history.

## Introduction

Lung cancer remains the leading cause of cancer-related mortality worldwide, with non-small cell lung cancer (NSCLC) representing the most common histological subtype, accounting for more than 80% of all lung cancer cases (1). While lung adenocarcinoma commonly metastasizes to sites such as the brain, bones, liver, and adrenal glands, prostatic metastasis is exceedingly rare, with only a few cases reported in the literature to date (2). Moreover, metastatic lung adenocarcinoma to the prostate without an identifiable primary lung lesion has never been documented, making such cases diagnostically challenging and clinically significant.

In recent years, advances in molecular genetics have revolutionized the management of NSCLC, with targeted therapy emerging as a cornerstone of treatment for patients harboring specific driver mutations (3). Among these, mutations in the epidermal growth factor receptor (EGFR) gene are the most prevalent, occurring in 45% of Asian patients and 20% of White patients with lung adenocarcinoma (4). For patients with sensitizing EGFR mutations, such as the exon 21 L858R mutation, EGFR tyrosine kinase inhibitors (TKIs) are recommended as first-line therapy due to their proven efficacy and favorable safety profile (4). Here, we present a unique case of a 64-years-old male with a history of hepatocellular carcinoma (HCC) treated surgically in 2014, who subsequently developed metastatic lung adenocarcinoma to the prostate without evidence of a primary lung lesion. This case exemplifies a “triple inversion” phenomenon, characterized by (1) the development of lung adenocarcinoma following HCC, (2) rare prostate metastasis, and (3) the identification of an EGFR exon 21 L858R mutation in the metastatic prostate lesion. Although no primary lung lesions were identified on imaging, lung adenocarcinoma was initially diagnosed based on histopathological and immunohistochemical findings from the prostate biopsy. Further molecular testing uncovered an EGFR L858R mutation, confirming the pulmonary origin and guiding the treatment strategy. The patient was initiated on Osimertinib, a third-generation EGFR tyrosine kinase inhibitor, which resulted in significant clinical improvement. This case highlights the importance of molecular profiling in diagnosing rare metastatic patterns and underscores the therapeutic potential of targeted therapies in complex oncological scenarios.

## Case presentation

**FIGURE 1:**
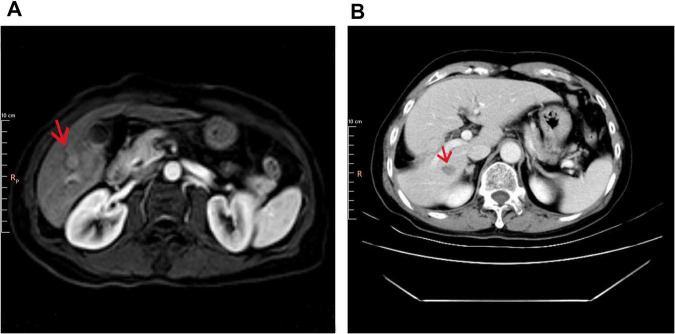
Imaging findings at baseline and post-operation. **(A)** Baseline liver imaging with abdominal MRI (T1 post-contrast, arterial phase). The arrow indicates mild liver cirrhosis and a 2 cm hypervascular mass in the right hepatic lobe (Segment VI). **(B)** Postoperative CT (Portal Venous Phase) showing surgical resection of liver segments V and VI.

A 64-years-old male with a history of chronic hepatitis B virus (HBV) infection presented to our institution in April 2014 after a liver mass was incidentally discovered during a routine health examination. The patient had a significant medical history, including a 30-years smoking habit (20 cigarettes per day) and a 30-years history of daily alcohol consumption (200 g per day). Upper abdominal magnetic resonance imaging (MRI) revealed mild liver cirrhosis and a small nodular lesion (2 cm in diameter) in the inferior segment of the right hepatic lobe, radiologically suggestive of hepatocellular carcinoma (HCC) (Figure 1A). Additionally, mildly enlarged lymph nodes were observed in the hepatic hilar region. Systemic imaging studies confirmed the absence of distant metastasis.

In May 2014, the patient underwent surgical resection of liver segments V and VI under general anesthesia (Figure 1B). Histopathological examination of the resected specimen confirmed the diagnosis of hepatocellular carcinoma. Immunohistochemical staining was positive for CK8/18, Heppar-1, GPC3, and GS, while negative for CK7, AFP, TTF-1, and SALL4. CD34 staining highlighted vascular structures, and CD56 was partially positive (Supplementary Figure 1). As assessed by Ki67, the proliferation index was low (1%–2%). Reticulin staining demonstrated reduced fibrous tissue, further supporting the diagnosis of HCC. Postoperatively, the patient was initiated on long-term antiviral therapy with entecavir dispersible tablets and remained under regular surveillance. No evidence of tumor recurrence or metastasis was observed during follow-up.

**FIGURE 2:**
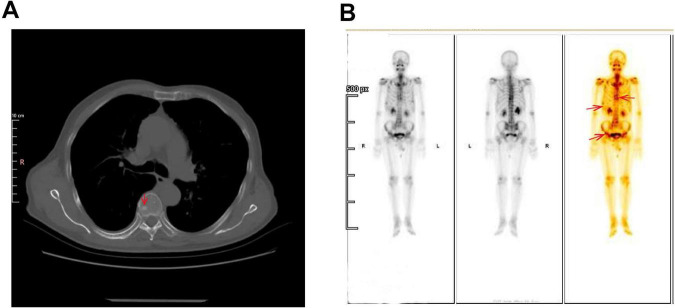
Imaging findings of suspected bone metastases. **(A)** Non-contrast CT scan of Chest and Abdomen (Bone Window). The circled area highlights the osteoblastic lesions in the bilateral ribs and partial vertebrae (thoracic, lumbar, and sacral regions), which raise suspicion for metastatic disease. These high-density lesions are indicative of bone metastasis. **(B)** Whole-body Tc-99m Methylene Diphosphonate (MDP) Bone Scintigraphy. A bone scan demonstrates multiple foci of increased radiotracer uptake in the thoracic spine, bilateral ribs, and pelvic bones (arrow). These findings are highly suggestive of osseous metastases.

In October 2022, during a routine follow-up visit, serum carcinoembryonic antigen (CEA) levels had risen to 27.44 ng/mL (normal range: 0–6 ng/mL), prompting an abdominal ultrasound that revealed prostate enlargement. Despite the elevated CEA, the patient remained asymptomatic and was advised to undergo further diagnostic evaluation, including advanced imaging and potential biopsy, to rule out occult malignancy or other causes of CEA elevation. However, the patient initially declined additional investigations and opted for continued surveillance. CEA levels continued to rise, reaching 45.86 ng/mL in April 2023 and peaking above 60 ng/mL by August 2023. These progressive elevations eventually led to further imaging studies in 2023. In March 2023, a non-contrast CT scan of the chest and abdomen revealed new high-density nodules in bilateral ribs and partial vertebrae (thoracic, lumbar, and sacral regions), raising suspicion for metastatic disease (Figure 2A). The liver showed partial absence of the right posterior lobe, consistent with the prior resection, and the prostate was noted to be enlarged with calcifications. By April 2023, the patient’s CEA level had further increased to 45.86 ng/mL, while alpha-fetoprotein (AFP) levels remained within normal limits. An upper abdominal MRI performed on April 8, 2023, confirmed the absence of the right posterior liver lobe but showed no evidence of HCC recurrence or metastasis.

A bone scan conducted in April 2023 revealed multiple areas of radiotracer uptake throughout the skeleton, highly suggestive of osseous metastases (Figure 2B). Moreover, magnetic resonance imaging (MRI) of the thoracic and lumbar spine on demonstrated multiple abnormal signal nodules in the vertebrae, consistent with metastatic involvement (Supplementary Figure 2). A gastrointestinal endoscopy was performed, and there was no definitive evidence of primary gastrointestinal malignancy.

**FIGURE 3:**
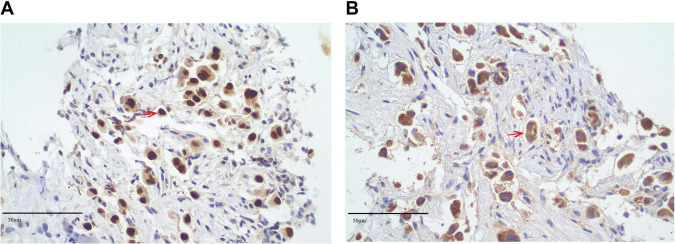
Immunohistochemical analysis of lumbar spine biopsy specimen. **(A)** Immunohistochemical staining for thyroid transcription factor-1 (TTF-1) shows strong nuclear positivity (TTF-1+, red arrows) in tumor cells, indicating a possible pulmonary origin (original magnification × 40; scale bar = 50 μm). **(B)** Immunohistochemical staining for Napsin A demonstrates cytoplasmic granular positivity (Napsin A+, red arrows) in the same tumor cells (original magnification × 40). TTF-1 and Napsin A are highly specific markers for lung adenocarcinoma, supporting the diagnosis of metastatic pulmonary adenocarcinoma involving the lumbar spine. All staining was performed using validated immunohistochemical protocols, and tissue sections were counterstained with hematoxylin. Positive and negative controls were included to confirm staining specificity.

In May 2023, a lumbar spine biopsy was performed. The histopathological examination of the lumbar spine biopsy revealed the presence of atypical cells, suggesting a malignancy. Immunohistochemical staining showed positive results for PCK, CK7, CK8/18, CK19, TTF-1, Napsin A, and Ki-67 (5%–10%), supporting the diagnosis of adenocarcinoma, likely of pulmonary origin. Negative markers, including CK20, CDX-2, SATB2, GATA3, PSA, PAX8, Heppar1, AFP, CD56, Synaptophysin, and CgA, helped rule out other primary malignancies such as gastrointestinal, prostate, hepatocellular, and neuroendocrine cancers. Immunohistochemical staining of the lumbar spine biopsy revealed positive results for TTF-1 (Figure 3A) and Napsin A (Figure 3B), which are highly specific markers for lung adenocarcinoma. TTF-1 exhibited strong nuclear positivity, while Napsin A demonstrated characteristic cytoplasmic granular staining. These dual marker findings strongly support the diagnosis of pulmonary adenocarcinoma. The presence of TTF-1 and Napsin A in combination is indicative of a lung-origin tumor, confirming the likely diagnosis of metastatic lung adenocarcinoma.

**FIGURE 4:**
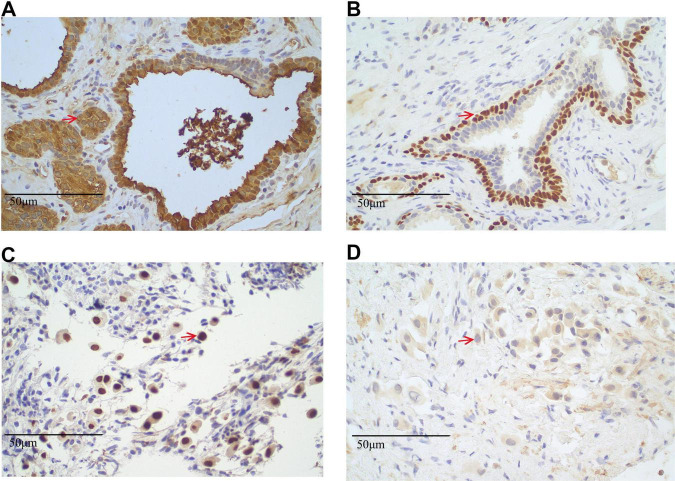
Immunohistochemical staining results of prostate biopsy specimens. **(A,B)** Left prostate tissue from Core 1. **(A)** Immunohistochemical staining for prostate-specific antigen (PSA) shows strong cytoplasmic positivity (PSA^+^), confirming prostatic origin. Positive cells are indicated by red arrows (original magnification × 40). **(B)** P63 immunostaining reveals distinct nuclear positivity (P63^+^) in basal cells, a feature of benign prostatic glands. Positive nuclei are marked by red arrows (original magnification × 40; scale bar = 50 μm). **(C,D)** Right prostate tissue from biopsy Cores 2 and 4. **(C)** Thyroid transcription factor-1 (TTF-1) staining shows nuclear positivity (TTF-1^+^) in atypical glandular cells, suggestive of metastatic lung adenocarcinoma. Positive cells are indicated by red arrows (original magnification × 40; scale bar = 50 μm). **(D)** PSA staining is negative (PSA^–^) in the same region, supporting a non-prostatic origin. Representative areas are marked with red arrows (original magnification × 40; scale bar = 50 μm). All sections were stained using standard immunohistochemistry protocols and counterstained with hematoxylin. Appropriate positive and negative controls were used.

These findings strongly suggest metastatic lung adenocarcinoma with spinal metastasis, warranting further investigation to assess the extent of disease spread. A PET-CT scan was performed in June 2023 to identify the primary lesion further. The findings included: (1) The PET-CT scan shows increased FDG uptake, predominantly consistent with osteoblastic (bone-forming) metastasis, a pattern commonly seen in prostate cancer metastasis. The maximum standardized uptake value (SUVmax) was 3.3, with other lesions exhibiting varying levels of FDG uptake, suggesting heterogeneous metastatic activity at different sites (predominantly osteoblastic) (Supplementary Figure 3); (2) a calcified nodule in the right peripheral zone of the prostate with increased FDG uptake (SUVmax 3.3), suggestive of prostate carcinoma, this lesion has abnormal metabolic activity, indicating the presence of malignancy, pending pathological confirmation (Supplementary Figure 4) and (3) postoperative changes in the liver with no evidence of hepatic lesions or increased FDG uptake. According to the PET-CT findings on June 2, 2023, the patient’s tumor was staged as lung adenocarcinoma TxN0M1c, stage IVB (8th edition of the TNM classification for NSCLC). To reduce the risk of skeletal-related events due to vertebral metastases, the patient received bone protection therapy with zoledronic acid 4 mg intravenously every 4 weeks. From December 2024 onward, the treatment was switched to denosumab 120 mg subcutaneously every 4 weeks for ongoing skeletal protection. No other malignant lesions were detected elsewhere in the body. Given the absence of malignant lesions in the liver and lungs, a prostate biopsy was performed. Histopathological examination of the prostate biopsy samples revealed that the tissue from the left prostate (Core 1) was consistent with benign prostatic hyperplasia (BPH). Immunohistochemical staining showed positive results for PSA (Figure 4A), P63 (Figure 4B), and CK5/6, which is characteristic of benign prostatic tissue, with no evidence of malignancy. In contrast, the biopsy samples from the right prostate (Cores 2 and 4) demonstrated minimal atypical cells. The immunohistochemical profile of these samples showed positive staining for TTF-1 (Figure 4C) and Ki67 but negative PSA (Figure 4D), suggesting a possible metastatic origin. The presence of TTF-1 positivity, a marker commonly associated with lung adenocarcinoma, supports the diagnosis of metastatic lung adenocarcinoma to the prostate. The lack of typical prostatic markers such as PSA, P63, and CK5/6 further strengthens the likelihood of a metastatic process rather than primary prostate carcinoma.

On June 29, 2023, genetic testing of the prostate specimen identified an EGFR exon 21 L858R mutation (Supplementary Table 1). Based on the identification of an EGFR exon 21 L858R mutation in the metastatic prostate lesion, the patient was started on osimertinib at the standard dose of 80 mg orally once daily. This treatment decision followed current NCCN guidelines for first-line therapy in EGFR-mutant advanced NSCLC (5). The patient demonstrated rapid biochemical and clinical improvement following osimertinib initiation (80 mg daily). The patient exhibited a marked biochemical response to therapy, as evidenced by a reduction in serum CEA levels from above 60 ng/ml on August 10, 2023, to within the normal reference range of 1.96 ng/mL by the follow-up on June 13, 2024. The patient has continued regular follow-up with chest and abdominal CT scans, as well as pelvic MRI, but no PET-CT was performed. The patient initiated Osimertinib therapy in late August 2023, which has been maintained without interruption. Serial evaluations of CEA levels remained within the normal range, with a value of 4.38 ng/mL observed on March 28, 2025. Radiological assessments throughout the treatment period consistently demonstrated stable disease. Clinically, the patient experienced marked improvement in functional status, with the Eastern Cooperative Oncology Group (ECOG) performance status improving from 2 to 0. The patient remains on Osimertinib therapy and is scheduled for clinical and radiological reassessment at the end of June 2025.

## Discussion

This case report describes a diagnostically challenging presentation of metastatic lung adenocarcinoma, marked by three consecutive diagnostic reversals. Initially, the patient’s history of hepatocellular carcinoma (HCC) and osseous metastasis suggested recurrence of HCC. However, immunohistochemical analysis (TTF-1+/Napsin A+) pointed to a pulmonary origin, despite the absence of radiographically visible lung lesions. The discovery of prostate metastasis, an exceedingly rare site for lung adenocarcinoma, further complicated the case and deviated from typical metastatic patterns.

Despite the absence of detectable primary lung lesions on imaging, the diagnosis of lung adenocarcinoma was first suggested through histopathological and immunohistochemical analysis of the prostate biopsy. Molecular profiling was performed to clarify the tumor origin and guide therapy further, revealing an EGFR exon 21 L858R mutation. This finding not only supported a pulmonary origin but also directly influenced clinical management by enabling the use of osimertinib, an EGFR-targeted therapy. Following the initiation of osimertinib, the patient achieved remarkable disease stabilization, highlighting molecular diagnostics’ critical role in the accurate classification and personalized treatment of atypical metastatic cancers. This case emphasizes the limitations of conventional diagnostic algorithms relying on clinical history and imaging and underscores the critical role of immunohistochemistry and molecular profiling in accurate diagnosis.

Metastatic carcinoma of unknown primary origin (CUP) accounts for approximately 3%–5% of all cancer diagnoses, presenting significant diagnostic challenges (6). Correct identification of the primary site is essential for effective treatment but is often hindered by non-specific clinical and imaging findings. In this case, the initial suspicion of prostate cancer was ruled out through comprehensive immunohistochemical (IHC) analysis, which revealed the absence of prostate-specific markers (PSA, NKX3.1) and the presence of lung-specific markers (TTF-1, Napsin A) in the prostate biopsy. This redirected the diagnosis to a pulmonary origin, even without identifiable lung lesions on imaging, highlighting the limitations of conventional imaging for detecting occult primary tumors.

Thyroid transcription factor-1 and Napsin A are critical immunohistochemical markers for identifying carcinomas of pulmonary origin (7). TTF-1, a nuclear protein encoded by the NKX2-1 gene, is highly specific for lung and thyroid carcinomas, while Napsin A, an aspartic protease, is specific for pulmonary adenocarcinomas (8, 9). Together, these markers have a combined sensitivity of 75%–85% for TTF-1 and 80%–90% for Napsin A in lung adenocarcinoma, providing essential diagnostic clarity when assessing cancers of unknown primary origin (8–10).

Molecular profiling, particularly identifying the EGFR exon 21 L858R mutation, reinforced the suspicion of lung adenocarcinoma as the primary source despite the absence of detectable primary lung lesions on imaging. This finding, alongside histopathological and immunohistochemical evidence, ultimately contributed to the diagnosis of metastatic lung adenocarcinoma. This mutation, commonly associated with lung cancer, resolved the diagnostic ambiguity and revealed a therapeutically actionable target. Consequently, the patient was treated with osimertinib, a third-generation EGFR-TKI, which resulted in a significant clinical improvement. The identification of the EGFR L858R mutation was pivotal in narrowing the differential diagnosis, especially given the unusual presentation without a detectable pulmonary lesion (11, 12). This molecular finding not only guided the diagnosis but also provided a rationale for initiating EGFR-targeted therapy, underscoring the importance of comprehensive molecular profiling in atypical metastatic presentations. Previous studies (13, 14) have consistently reported that the presence of the L858R mutation in the EGFR gene correlates with improved responsiveness to EGFR tyrosine kinase inhibitors (TKIs) such as gefitinib, erlotinib, and afatinib, as observed in patients with metastatic NSCLC. This is in line with our findings, where the mutation’s identification suggests the potential for EGFR TKI therapy as a first-line treatment, as these therapies can block the constitutive activation of EGFR signaling, leading to reduced tumor proliferation and enhanced patient survival. The National Comprehensive Cancer Network (NCCN) guidelines recommend EGFR-TKI therapy for patients with EGFR mutations, as it significantly improves progression-free and overall survival in EGFR-mutant non-small cell lung cancer (NSCLC) (5). In this case, osimertinib was chosen due to its proven efficacy in treating EGFR-mutant NSCLC, particularly in cases with atypical metastatic sites (15). Furthermore, the National Medical Products Administration (NMPA) of China has approved additional EGFR-TKIs, including icotinib, almonertinib, and furmonertinib, for the treatment of EGFR-mutant NSCLC (16). The tumor was classified as microsatellite stable (MSS), suggesting limited benefit from immune checkpoint inhibitors (ICIs) based on current evidence. Further assessment of PD-L1 expression and tumor mutational burden (TMB) may provide additional insights into the potential role of immunotherapy. In cases where targeted therapy is not feasible, chemotherapy remains a viable option, with agents such as cisplatin and docetaxel being the preferred options for advanced NSCLC. The choice of chemotherapy should be individualized, considering the patient’s clinical status and overall treatment goals.

Prostatic metastasis from lung adenocarcinoma is extremely rare, with only a limited number of cases reported. A PubMed search identified four cases of lung adenocarcinoma metastasizing to the prostate (Table 1) (17–20). These cases typically involved patients with known pulmonary lesions and symptoms such as urinary difficulty or elevated PSA. Most diagnoses were confirmed via prostate biopsy demonstrating TTF-1 and CK7 positivity. Unlike previously reported cases, our patient had no identifiable pulmonary lesion on imaging, yet prostate biopsy revealed histological and immunohistochemical features consistent with lung adenocarcinoma. Molecular analysis detected an EGFR exon 21 L858R mutation, supporting the diagnosis of metastatic lung adenocarcinoma. The patient responded favorably to osimertinib, highlighting the importance of molecular profiling in such atypical presentations.

**TABLE 1:** Reported cases of lung adenocarcinoma metastasis to the prostate.

References	Age/sex	Primary lung site and stage	Molecular/IHC profile	Metastatic sites	Diagnostic method	Treatment	Outcome
Present case (2025)	64/M	No lesion on imaging; EGFR + lung adenoca	EGFR exon 21 L858R+, TTF-1+, CK7+	Prostate, bone	Prostate biopsy, IHC, EGFR mutation test	Osimertinib (targeted therapy)	Favorable response
Gilmour et al. (17)	55/M	Left upper lobe, T2 N3 M1	EGFR-, ALK-	Brain, bone (T2), adrenals, lungs	Imaging, biopsy	Chemo, radiation, atezolizumab	Progressed with widespread metastasis
Kamel et al. (18)	72/M	Right middle lobe, stage IA2 (cT1bN0M0)	EGFR-, PD-L1 < 1%; IHC: TTF-1+, CK7+, AE1/3+, NKX3-	Isolated prostate	PET/CT, prostate biopsy with IHC	SBRT to lung and prostate; lymphoma therapy	Remission at 1 year
Shi et al. (19)	73/M	Right upper lobe, prior lobectomy	TTF-1+, CK7+, PSA-	Cervical lymph node, prostate	PSA elevation, prostate biopsy, IHC	Radiotherapy (to lymph node); refused biopsy	Discharged stable; urinary symptoms resolved
Yu et al. (20)	59/M	Pulmonary mucinous adenocarcinoma	PSA-, TTF-1+, CK7+, CK20 (partial+), Villin+, CDX-2+, Ki-67 (70%), P63+, CK34βE12+; P504S-	Prostate	MRI, CT, TURP pathology, IHC	TURP; no systemic treatment reported	Urinary symptoms resolved; discharged stable

CT, computed tomography; CYFRA 21-1, cytokeratin-19 fragment; DWI, diffusion-weighted imaging; EGFR, epidermal growth factor receptor; HE, hematoxylin and eosin; HTLV-1, human T-cell leukemia virus type 1; IHC, immunohistochemistry; IMRI, magnetic resonance imaging; PET/CT, positron emission tomography–computed tomography; PSA, prostate-specific antigen; SBRT, stereotactic body radiation therapy; tPSA, total prostate-specific antigen; TTF-1, thyroid transcription factor-1; TURP, transurethral resection of the prostate.

Although the exact mechanism occurrence of prostate metastasis from lung adenocarcinoma remains unclear, possible explanations include vascular tropism through Batson’s plexus (21) or interaction between EGFR and androgen receptor (AR) signaling pathways (22). This case highlights the complexity of metastatic patterns and the need for further investigation into the mechanisms driving such rare occurrences. However, it is important to acknowledge that these mechanistic hypotheses remain speculative in the context of our case, as no direct molecular or pathological evidence was obtained to confirm the involvement of these pathways. Although prostate cancer typically produces elevated PSA levels, the patient’s PSA levels remained normal throughout the treatment process, ruling out prostate cancer as the primary tumor. Immunohistochemical analysis of the prostate lesion also showed the absence of PSA, which further confirmed the diagnosis of metastatic lung adenocarcinoma. The identification of the EGFR L858R mutation enabled the use of targeted therapies, such as gefitinib, erlotinib, and osimertinib, which have been shown to improve survival in EGFR-mutant NSCLC. Our findings in this case report align with existing literature demonstrating that first-line EGFR tyrosine kinase inhibitors (TKIs), including osimertinib, almonertinib, and furmonertinib, offer durable responses in patients with EGFR-mutant NSCLC. The FLAURA trial demonstrated that osimertinib significantly improved median progression-free survival (PFS) (18.9 months vs. 10.2 months) compared to first-generation TKIs, with an associated decline in tumor biomarkers (13). Similarly, studies on almonertinib and furmonertinib have reported promising efficacy, particularly in patients harboring the EGFR exon 19 deletion or L858R mutation (23, 24). In contrast, the MSS status of the tumor suggests limited benefit from ICIs, highlighting the need for additional biomarkers, such as PD-L1 expression and TMB, to assess the suitability of immunotherapy. Previous studies, including the KEYNOTE-189 trial (25) and IMpower150 trial (26), have reported that EGFR-mutant MSS tumors exhibit poor response to PD-1/PD-L1 blockade. This may be attributed to the low tumor mutational burden (TMB) and an immunosuppressive tumor microenvironment. For patients who are not candidates for targeted or immunotherapy, chemotherapy remains an alternative (4).

This case underscores the importance of a multidisciplinary approach in managing complex metastatic presentations, integrating clinical, imaging, histopathological, and molecular data to optimize diagnostic accuracy and therapeutic decisions. The absence of a primary lung lesion on imaging further emphasizes the indispensable role of advanced molecular techniques in modern oncologic practice. One of the key lessons from this case is the importance of considering the possibility of a new primary malignancy in patients with a history of cancer. The initial assumption of HCC recurrence based on the patient’s history and elevated CEA levels highlights the potential for diagnostic anchoring bias. Additionally, the limited sensitivity of PET-CT for detecting small lung lesions emphasizes the need for complementary diagnostic tools, including molecular profiling, in challenging cases. From a therapeutic standpoint, the patient’s favorable response to osimertinib reaffirms the value of precision oncology in managing complex metastatic diseases. This case highlights the importance of EGFR mutation status in guiding treatment decisions, even when metastatic spread occurs to uncommon sites such as the prostate. This case also has broader implications for clinical practice, emphasizing the need for thorough molecular profiling in patients with rare or unusual metastatic patterns. It also advocates for a comprehensive approach to patient care that considers second malignancies, particularly when unusual metastatic patterns or unexplained symptoms are present. Further research should focus on understanding the mechanisms of rare metastases, such as prostate involvement, to optimize surveillance and treatment protocols for patients with complex cancer histories.

This study has several limitations. First, a bronchoscopy was not performed to confirm the pulmonary origin of the tumor further, as the patient declined the procedure despite counseling. Second, an enhanced MRI of the head was not conducted to evaluate potential brain metastases due to the patient’s decision to forgo additional imaging, considering his advanced disease stage and absence of neurological symptoms. Lastly, while TNM staging was included based on PET-CT findings and the 8th edition classification, the latest 9th edition was not applied as it was not yet implemented at the time of diagnosis. These factors may limit the comprehensiveness of the diagnostic evaluation.

## Patient perspective

This patient had been managing his past medical conditions when he developed new health concerns. He experienced unexplained fatigue and mild pelvic discomfort, attributing them to his liver issues. During a routine check-up, elevated CEA levels and imaging raised suspicion of metastatic disease. Bone scan and PET-CT revealed high-density lesions in the prostate, leading the patient to suspect prostate cancer. However, biopsy results were surprising, showing poorly differentiated carcinoma, and immunohistochemistry suggested a lung cancer origin. The patient struggled to understand how lung cancer could metastasize to his prostate without visible signs of primary lung lesions. The diagnosis was confusing, as he had been focused on managing his HCC. Molecular testing confirmed an EGFR exon 21 L858R mutation, strengthening the diagnosis of metastatic lung adenocarcinoma. Initially apprehensive about treatment, the patient began Osimertinib therapy and noticed gradual improvement. Over time, the patient felt reassured by the therapy’s effectiveness and appreciated the personalized approach, feeling supported by his medical team and grateful for improved quality of life.

## Conclusion

In conclusion, this case illustrates the importance of a comprehensive, multidisciplinary approach in managing metastatic carcinoma of unknown primary origin. Advances in immunohistochemistry and molecular diagnostics have significantly altered the therapeutic landscape for lung adenocarcinoma. Targeted therapies, when appropriate, offer substantial improvements in patient outcomes. However, further evaluation of immunotherapy and chemotherapy remains essential for cases where targeted therapy is not feasible. As demonstrated, careful integration of clinical, imaging, histopathological, and molecular data is crucial in optimizing treatment strategies for patients with complex metastatic presentations.

## Data Availability

The original contributions presented in this study are included in this article/Supplementary material, further inquiries can be directed to the corresponding author.
