# Supplementary material for: Triple reversal phenomenon in EGFR-mutant lung adenocarcinoma with prostate metastasis following hepatocellular carcinoma: a rare Case Report with diagnostic and therapeutic implications
Source: Front Med (Lausanne). 2025 Jul 16;12:1619466. doi: 10.3389/fmed.2025.1619466 (PMC12307205; doi:10.3389/fmed.2025.1619466)
Supplement: Supplementary file 1 [file Supplementary_file_1.docx]

**Triple Reversal Phenomenon in EGFR-Mutant Lung Adenocarcinoma with Prostate Metastasis Following Hepatocellular Carcinoma: A Rare Case Report with Diagnostic and Therapeutic Implications**

Jieyan Luo^1^, Jie Zhou^1^, Li Liu^1^*

^1^Cancer Center, Ziyang Central Hospital, No. 66, Rende West Road, Yanjiang District, Ziyang City, Sichuan Province, 641300, China.

***Corresponding author**: Dr. Li Liu, Cancer Center, Ziyang Central Hospital, No. 66, Rende West Road, Yanjiang District, Ziyang City, Sichuan Province, 641300, China. E-mail: [764609577@qq.com](mailto:764609577@qq.com)

**Supplementary Figure S1**: Immunohistochemistry (IHC) of resected hepatocellumar carcinoma (HCC). (**A**) Immunohistochemical staining of GPC3. (**B**) Immunohistochemical staining of HepPar-1. (**C**) Immunohistochemical staining of Reticulin Loss. This figure shows the immunohistochemistry (IHC) staining of the resected liver tissue from segments V and VI, confirming the diagnosis of hepatocellular carcinoma (HCC). The IHC results demonstrate strong positivity for GPC3 and HepPar-1, markers commonly associated with HCC. Additionally, reticulin loss is observed, further supporting the diagnosis of HCC in the resected specimen. Note: IHC was performed using standard protocols for GPC3, HepPar-1, and reticulin (Scale bar: 100 µm).


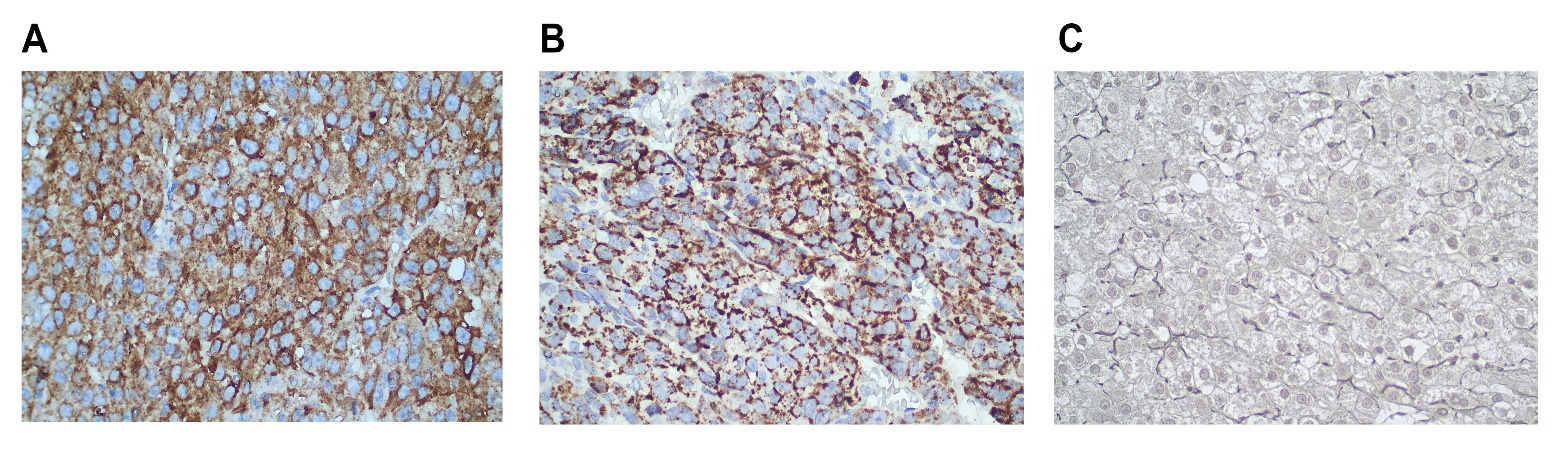


**Supplementary Figure S2**: Sagittal T1-weighted MRI of the thoracolumbar spine demonstrating osteoblastic metastases. The midline slice demonstrates hypointense osteoblastic metastases (red circle) in the T12 and L2 vertebrae, indicating osteoblastic involvement. These findings are consistent with metastatic lesions, further supporting the diagnosis of osseous metastasis.


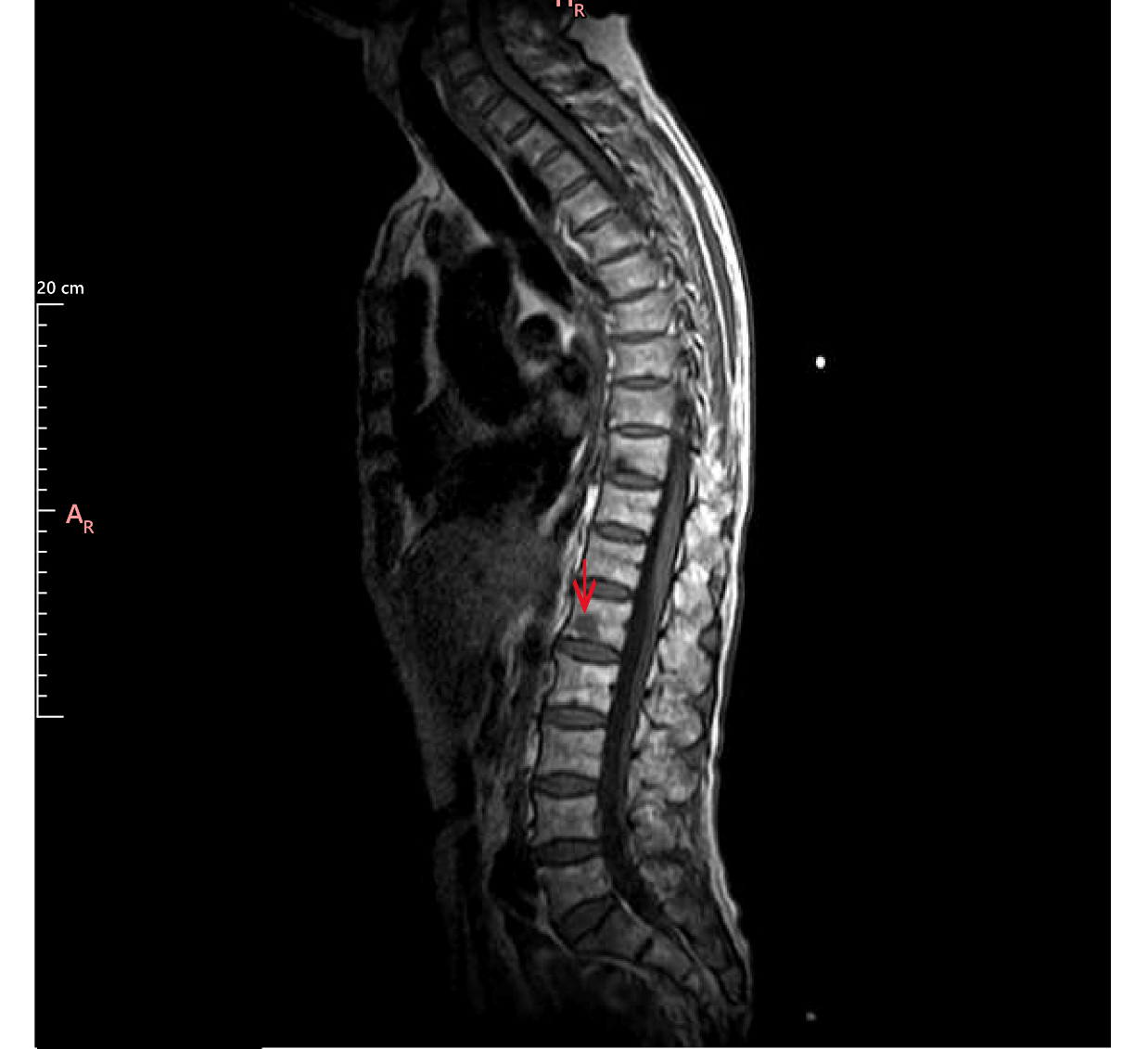


**Supplementary Figure S3**: PET-CT scan showing increased FDG uptake consistent with osteoblastic (bone-forming) metastasis. The pattern of metastasis is typically associated with prostate cancer, with a maximum standardized uptake value (SUVmax) of 3.3. Other lesions exhibit varying levels of FDG uptake, indicating heterogeneous metastatic activity predominantly osteoblastic.


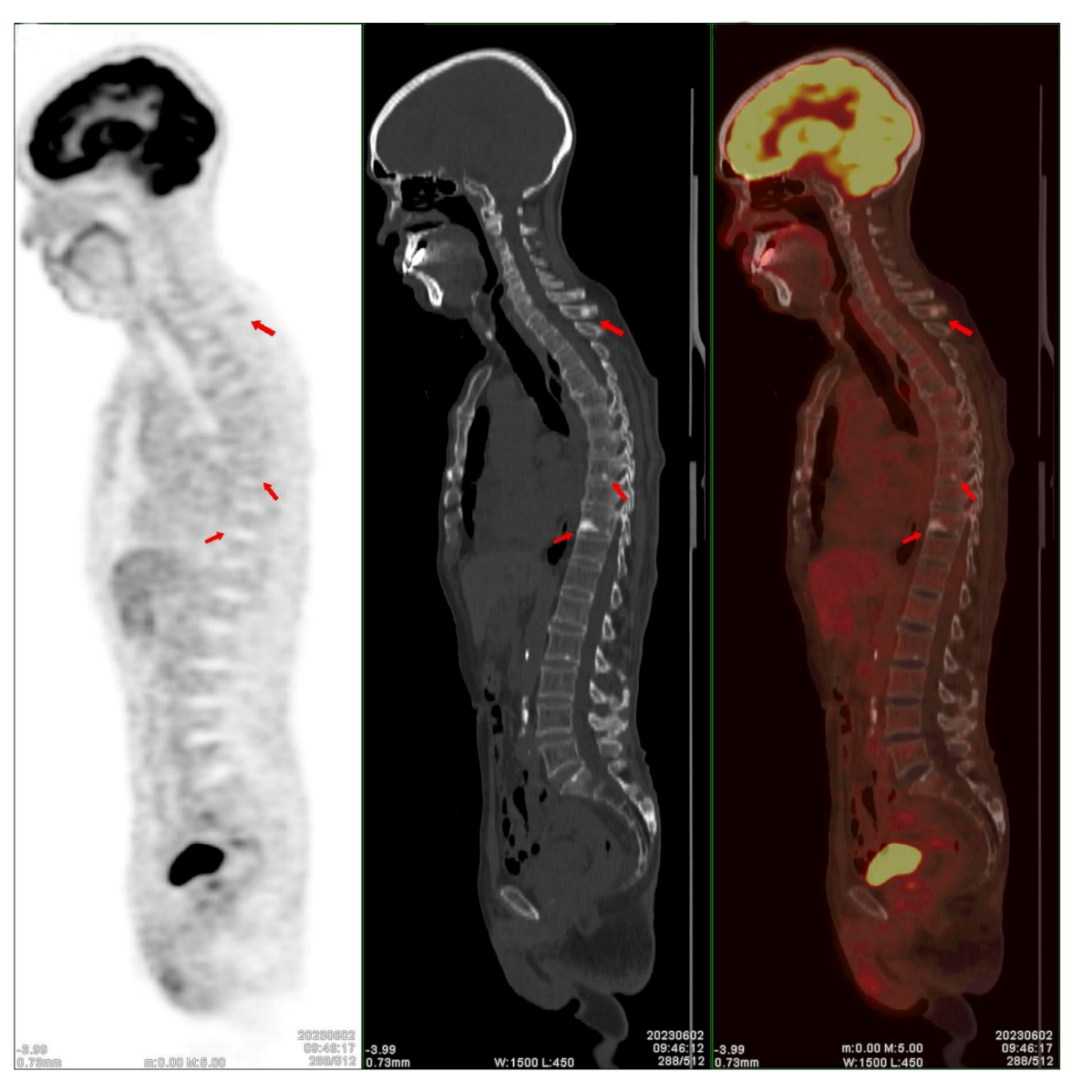


**Supplementary Figure S4**: PET-CT scan showing a calcified nodule in the right peripheral zone of the prostate with increased FDG uptake (SUVmax 3.3), suggestive of prostate carcinoma. The abnormal metabolic activity of this lesion raises suspicion for malignancy, pending pathological confirmation.


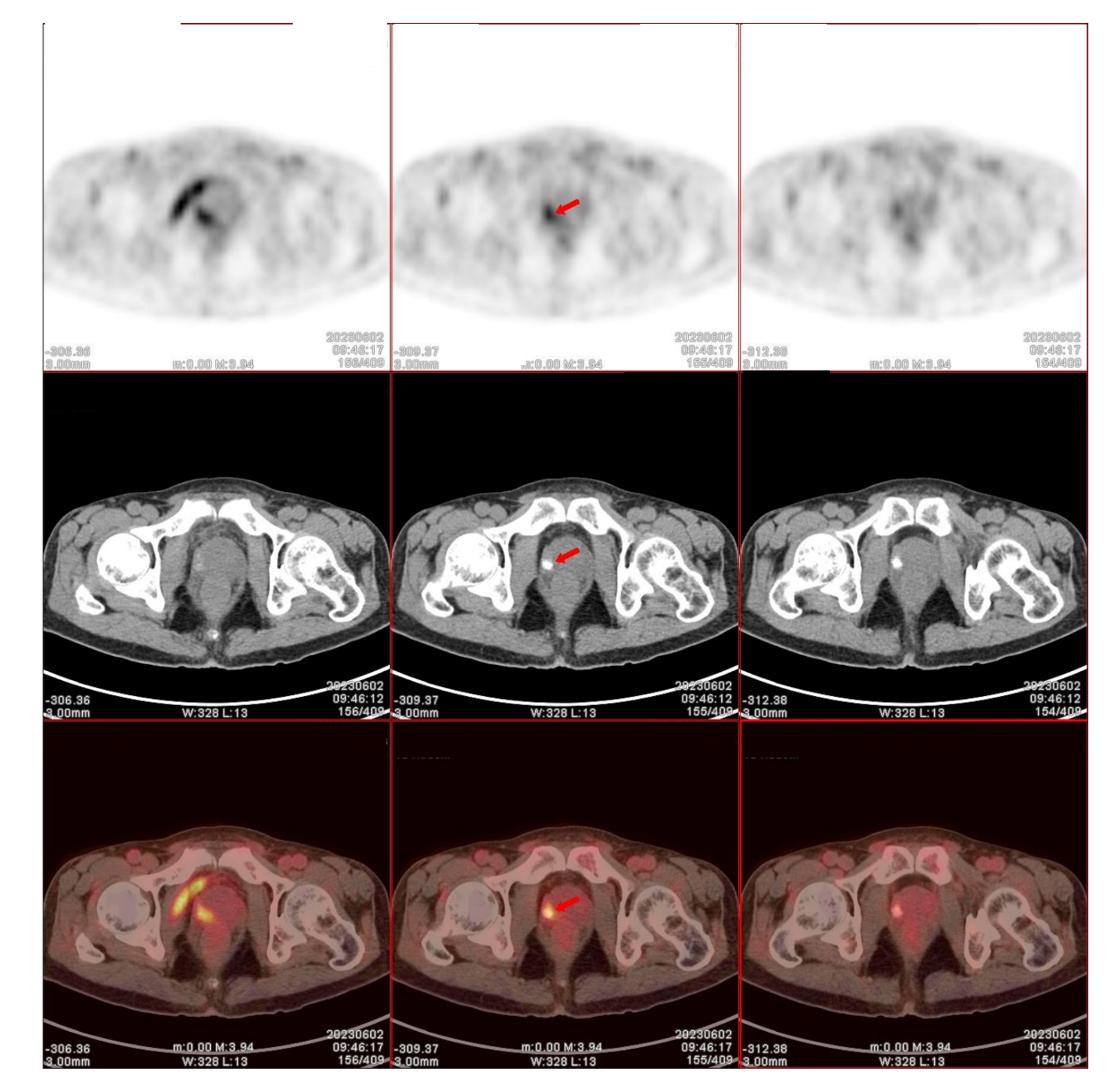


**Supplementary Table S1**. Summary of Genetic testing and treatment test Results of the patient.

| **Test** | **Result** | **Interpretation** | **Treatment Recommendations** |
| --- | --- | --- | --- |
| **EGFR Gene Exon 21 Mutation** | L858R Mutation | The patient carries the EGFR L858R mutation, a common mutation in NSCLC. | **First-line EGFR-TKIs**: Gefitinib, Erlotinib, Afatinib, Osimertinib, Dacomitinib **NMPA-approved treatments**: Icotinib, Amitinib, Fumitib |
| **Microsatellite Instability (MSI)** | MSS (Microsatellite Stable) | The patient has MSS, which indicates a limited response to immune checkpoint inhibitors. | Further assessment of **PD-L1** expression and Tumor Mutational Burden is recommended to evaluate the potential benefit of immune therapy. |
| **Preferred Chemotherapy Drugs** | Cisplatin, Docetaxel | These are preferred chemotherapy agents for NSCLC treatment. | Use as first-line chemotherapy. |
| **Alternative Chemotherapy Drugs** | Oxaliplatin, Vincristine, Cyclophosphamide, Leucovorin, Raltitrexed, Gemcitabine, Fluorouracil, Capecitabine, Tegafur, Methotrexate, Pemetrexed, Vinorelbine, Paclitaxel, Doxorubicin, Epirubicin, Pirubicin, Mitomycin, Nedaplatin, Lobaplatin | These chemotherapy agents can be considered if preferred drugs are contraindicated or ineffective. | Consider as backup options for chemotherapy based on patient tolerance and specific clinical needs. |

NMPA: National Medical Products Administration; TKIs: Tyrosine Kinase Inhibitors; PD-L1: Programmed Death-Ligand 1
